# The possible “calming effect” of subchronic supplementation of a standardised phospholipid carrier-based *Melissa officinalis L*. extract in healthy adults with emotional distress and poor sleep conditions: results from a prospective, randomised, double-blinded, placebo-controlled clinical trial

**DOI:** 10.3389/fphar.2023.1250560

**Published:** 2023-10-19

**Authors:** Aasiya Bano, Piril Hepsomali, Fazle Rabbani, Umer Farooq, Ayesha Kanwal, Aisha Saleem, Ali Akbar Bugti, Aftab Alam Khan, Zainab Khalid, Mahroo Bugti, Shah Mureed, Saeed Khan, Ikram Din Ujjan, Sümeyye Şahin, Mehtap Kara, Amjad Khan

**Affiliations:** ^1^ PEOC, Department of Health, Quetta, Balochistan, Pakistan; ^2^ School of Psychology, University of Roehampton, London, United Kingdom; ^3^ Department of Psychiatry, Lady Reading Hospital, Peshawar, Pakistan; ^4^ Ayub Medical College and Teaching Hospital, Abbottabad, Pakistan; ^5^ Department of General Surgery, Bolan Medical Complex Hospital (BMCH), Quetta, Pakistan; ^6^ Department of Gynaecology and Obstetrics, BMCH, Quetta, Pakistan; ^7^ Department of Paediatrics, BMCH, Quetta, Pakistan; ^8^ Department of Pathology, Dow University of Health Sciences, Karachi, Pakistan; ^9^ Department of Pathology, Liaquat University of Medical and Health Sciences (LUMHS), Jamshoro, Pakistan; ^10^ Department of Food Engineering, Ordu University, Ordu, Türkiye; ^11^ Department of Pharmaceutical Toxicology, Faculty of Pharmacy, Istanbul University, Istanbul, Türkiye; ^12^ Nuffield Division of Clinical Laboratory Sciences, Radcliffe Department of Medicine, University of Oxford, Oxford, United Kingdom; ^13^ Department of Biochemistry, LUMHS, Jamshoro, Pakistan

**Keywords:** *Melissa officinalis L.,* lemon balm, depression, anxiety, stress, insomnia, γ-amino butyric acid transaminase, Phytosome^™^, Relissa^™^

## Abstract

**Background:** Emotional distress conditions such as depression, anxiety, stress, and poor sleep are widespread health problems that have a significant impact on people’s lives. Conventional drugs are commonly prescribed to treat emotional distress and poor sleep conditions; however, these medications have several limitations and have shown multiple side effects. Over recent years botanicals-based pharmacological agents have gained increasing research and clinical interest in the management of emotional distress and sleep disorder. Of note, *Melissa officinalis L.* (MO) leaf extract has demonstrated considerable neuropharmacological properties both in animal and human studies and has emerged as a promising natural “calming agent.” However, research in this area is limited, and more studies are needed to validate its efficacy in amelioration of emotional distress and poor sleep conditions.

**Objectives:** We aimed to assess the pharmacological effects of subchronic supplementation of an innovative standardised phospholipid carrier-based MO aqueous extract on emotional distress and poor sleep conditions.

**Design:** A 3-week prospective, randomised, placebo-controlled, parallel-group, double-blinded clinical trial was conducted in 100 healthy adults complaining of a moderate degree of depression, anxiety, or stress, with scores of ≥14, ≥10, and ≥19, respectively, in the self-report Depression, Anxiety, and Stress Scale (DASS-42) or poor sleep, as indicated by the score of >5 in the Pittsburgh Sleep Quality Index (PSQI) scale. In addition, the impact of emotional distress and/or poor sleep on participants’ mental wellbeing, emotional feelings, and quality of life was also assessed using the self-reported Warwick–Edinburgh Mental Wellbeing Scale (WEMWBS), Positive and Negative Affect Schedule (PANAS) scale, and quality of life (WHO-QoL-BREF) scale, respectively.

**Results:** Oral supplementation of 200 mg of phospholipid-based MO aqueous extract (Relissa™) tablets twice a day (i.e., 400 mg/day) for 3 weeks led to significant improvements in the depressive mood, anxiety, stress, positive and negative affect (emotional feelings), overall mental wellbeing, and quality-of-life scores (all *p* values <0.001). Supplementation of MO extract was well tolerated, and no treatment-emergent effects or serious adverse events were reported.

**Conclusion:** According to the results of this study, the phospholipid carrier-based MO aqueous extract possesses considerable neuropharmacological properties, and its supplementation may provide a promising therapeutic option for the management of moderate emotional distress and/or poor sleep conditions.

**Clinical Trial Registration:** clinicaltrials.gov, identifier NCT05602688.

## 1 Introduction

Emotional distress conditions, such as depression, stress, anxiety, and insomnia (sleep disorder), are the most prevalent mental health conditions affecting a considerable amount of people worldwide (the prevalence of sleep problems ranges from 5.0% to 50.0% (Morin and Jarrin, 2022), while depressive and anxiety disorders affect more than 280 million and 300 million people, respectively (World Health Organization, 2022), causing significant disruption to everyday life, affecting performance in occupational and educational settings, and placing considerable burden (trillions of dollars) on healthcare systems (Hafner et al., 2017; Hillman et al., 2018). It is also important to note that the COVID-19 pandemic has been found to trigger a 25% increase in the prevalence of anxiety and depression worldwide (Kupcova et al., 2023). Conventional drug treatment for depressive conditions involves the use of tricyclic antidepressants (TCAs), monoamine oxidase inhibitors (MAOIs), selective serotonin reuptake inhibitors (SSRIs), serotonin and norepinephrine reuptake inhibitors (SNRIs), norepinephrine–dopamine reuptake inhibitors (NDRIs), and serotonin antagonists and reuptake inhibitors (SARIs) (Arroll et al., 2016), while for anxiety disorders, the drug therapies include SSRIs, SNRIs, pregabalin, TCAs, buspirone, benzodiazepines (BZDs), and MAOIs (Bandelow et al., 2022). However, published data suggest that 30%–60% of people do not comply with these synthetic antidepressant or anxiolytic medications due to adverse events or a signatory delay in effectiveness (Demyttenaere, 2001). According to various studies, most of the conventional drugs used for emotional distress are associated with multiple side effects, such as cognitive dysfunction, excessive sedation, sexual dysfunction, respiratory depression, withdrawal syndrome, seizures, and suicide, caused by prolonged drug use or high drug resistance (de Niet et al., 2009; Seldenrijk et al., 2015; Seldenrijk et al., 2017). Conversely, these were reduced in 45% of the reported studies where dietary supplements were used as adjuvant for the same indications (Yeung et al., 2018). Over the past decades, exploration in botanical psychopharmacology has received considerable attention due to its efficacy, excellent safety, and tolerability. With growing scientific evidence, physicians and patients have developed an interest in the use of botanical-based pharmacological therapies (the use of some of them is supported by the European Medicines Agency herbal monograph (European Medicines Agency, 2023)) for the management of emotional distress and poor sleep conditions (Head and Kelly, 2009; Kavan et al., 2009; Liu et al., 2015; Kenda et al., 2022).

Of note, amongst the several reported botanicals for psychopharmacology, the leaf extract of *Melissa officinalis L.* (MO), commonly known as lemon balm mint, has emerged as a promising natural “calming agent” (Sharifi-Rad et al., 2021) for emotional distress and related conditions. The main chemical constituents responsible for the MO pharmacological effects are reportedly diverse antioxidant polyphenols (with rosmarinic acid (RA) as the major compound (>5%) and flavonoids, e.g., luteolin-3′-*O*-glucuronide) and terpenes (with citronellal, neral, and geranial as the most abundant constituents), among others (Uritu et al., 2018). *Melissa officinalis L.* extract is one of the most popular and widely used dietary supplements in central and southern Europe, the Mediterranean region, the United States, and South and West Asia. Historically, the therapeutic use of MO dates back over 2000 years through the Greeks and the Romans and is mentioned in the *Historia Plantarum* (approximately 300 B.C.) as “honey leaf”. For centuries, MO has been used in traditional Chinese medicine (TCM), traditional Persian medicine (TPM), Ayurvedic medicine, Arabic medicine, and medieval European medicine for treating diverse health conditions, such as insomnia, migraines, neuroses, and hysteria, among others (Shakeri et al., 2016). In the modern pharmacological studies, various human clinical trials (Kennedy et al., 2002; Akhondzadeh et al., 2003; Kennedy et al., 2003; Kennedy et al., 2004; Kennedy et al., 2006; Cases et al., 2011; Alijaniha et al., 2015; Ranjbar et al., 2018a; Ranjbar et al., 2018b; Haybar et al., 2018; Heydari et al., 2018; Soltanpour et al., 2019; Araj-Khodaei et al., 2020; Noguchi-Shinohara et al., 2020; Shirazi et al., 2021), animal models (Coleta et al., 2001; Guginski et al., 2009; Ibarra et al., 2010; Yoo et al., 2011; Taiwo et al., 2012; Feliú-Hemmelmann et al., 2013; Lin et al., 2015; Ghazizadeh et al., 2020; Talebi et al., 2022), and *in vitro* studies (Kennedy et al., 2003; Awad et al., 2007; Awad et al., 2009; López et al., 2009; Sahin et al., 2016) have investigated the neurotherapeutic effects of MO extract, with most studies reporting its antidepressant, anti-stress, anxiolytic, anti-insomnia, and anti-oxidative stress (neuroprotective) properties. According to reported evidence, the neuropharmacological effects of MO extract are due to its various biological properties, i.e., its GABAergic properties (upregulation of γ-amino butyric acid (GABA) through GABA-transaminase (GABA-T) inhibition (Awad et al., 2007; Awad et al., 2009; Yoo et al., 2011), GABA_A_ receptor affinity (Salah and Jäger, 2005; Abuhamdah et al., 2008; Sahin et al., 2016)), modulation of the serotonergic pathway (Dimpfel and Suter, 2008; Lin et al., 2015), and inhibition of acetylcholine esterase (AChE) (Perry et al., 1996; Ferreira et al., 2006; Dastmalchi et al., 2009) and monoamine oxidase (MAO) enzymes (Ulbricht et al., 2005; López et al., 2009; Taiwo et al., 2012; Shakeri et al., 2016). Some other studies have suggested that the neuropharmacological mechanism of MO extract may be due to its direct nicotinic and muscarinic cholinergic receptor binding properties (Perry et al., 1996; Wake et al., 2000; Kennedy et al., 2003) and its ability to decrease corticosterone levels (Yoo et al., 2011; Feliú-Hemmelmann et al., 2013). *Melissa officinalis L* extract also possesses strong antioxidant properties and helps in the protection of neuronal cells against oxidative stress damage (López et al., 2009; Miraj et al., 2017).

However, therapeutic evidence for MO’s neuropharmacological effects is still limited, and the determination of its optimal clinical dosage and duration remains unknown (Świąder et al., 2019). Further studies are needed to validate the efficacy of MO extract supplementation in conditions of depression, mood, anxiety, stress, and related conditions and to demonstrate its mechanism of action, safety, and tolerability. Moreover, many phytochemicals have limited bioavailability and bioactivity after ingestion due to their limited solubility, stability, and absorption characteristics. Hence, efforts should also be focused on developing efficient carrier systems to achieve improved MO bioavailability for physiological functions. In the present prospective, randomised, double-blinded, placebo-controlled clinical trial, for the first time, we investigated the possible neuropharmacological effects of an innovative phospholipid carrier-based (Phytosome™) MO aqueous extract (Relissa™) in individuals with a moderate degree of emotional distress and/or poor sleep conditions. It is anticipated that the phospholipid carrier will aid in achieving the maximum bioavailability of the extract chemical constituents for pharmacological MO effects.

## 2 Methods

### 2.1 Study participants

Participants were enrolled at Ayub Teaching Hospital, Abbottabad, Pakistan (PK); Lady Reading Hospital, Peshawar, Pakistan; and Bolan Medical Complex Hospital, Quetta, Pakistan, either at the hospital walk-in outpatients’ clinics or through community-based primary healthcare clinics from 3 January 2023 to 28 February 2023. The inclusion criteria were as follows: healthy adults, age: 18–65 years, of either gender, and with a moderate degree of depression, anxiety, or stress, i.e., with scores of ≥14, ≥10, and ≥19, respectively, in the self-report Depression, Anxiety, and Stress Scale (DASS-42) or poor sleep quality, i.e., score >5 in the Pittsburgh Sleep Quality Index (PSQI) scale. The exclusion criteria were as follows: current use of prescribed conventional medication or supplements for neuropsychiatric or severe sleep disorders; known history of neuropsychiatric or severe sleep disorders; history of any allergic reactions/hypersensitivity to MO extract or its constituent compounds; pregnant or lactating women, current use or history of illicit substance misuse; hypertension (systolic blood pressure ≥140 mmHg or diastolic blood pressure ≥90) or other cardiovascular diseases; body mass index (BMI) <18.5 and >29.9 kg/m^2^; hyperthyroidism, diabetes mellitus (DM), or cancer; use of blood thinner medications such as Coumadin (warfarin) or Plavix (clopidogrel), glaucoma medications like Travatan (travoprost), and chemotherapy drugs like tamoxifen and Camptosar (irinotecan); or any other condition or factor that in the opinion of the treating consultant contraindicates the use of MO extract for the participant.

The study was approved by the Institutional Ethics Review Committee of Ayub Teaching Hospital, Abbottabad, Pakistan (PK) (Ref. No. RC-/EA-01/194); Lady Reading Hospital, Peshawar, PK (Ref. No. 684/LRH/MTI); and Bolan Medical Complex Hospital, Quetta, PK (Ref. No. 5501). The study was carried out in accordance with the guidelines of the Declaration of Helsinki and Good Clinical Practice standards, and all participants provided informed written consent. The study was registered at clinicaltrials.gov, identifier NCT05602688.

### 2.2 Study design and treatment

This was a prospective, multi-centre, double-blinded, placebo-controlled, randomised clinical trial consisting of two arms: the MO phospholipid extract supplement and the placebo. By using G*Power, the sample size was estimated to be 50 subjects per group based on the results of the previous study, with a confidence interval of 0.95, a test power of 80%, and 25% sample attrition (Soltanpour et al., 2019). The anxiety score was utilised as a primary measure with an effect size of 0.97. The participants received the MO phospholipid extract supplement orally as a 200 mg tablet of Relissa™ (manufacturer: Indena S.p.A., Milan, Italy), taken at home twice a day after meals for 3 weeks. Relissa™ is an MO leaf aqueous extract that has been standardised to 17%–23% hydroxycinnamic acid derivatives; it is analysed for its rosmarinic acid content and formulated with the phospholipid (sunflower) (Phytosome™) carrier for improved bioavailability. Placebo tablets were indistinguishable in appearance from Relissa™ tablets and were used in the same way as Relissa™.

### 2.3 Clinical measures

Participants were screened for enrolment in the study using either the DASS-42 or PSQI questionnaire (provided in hard copies to the participants by a member of the clinical team) according to the required scores in the inclusion criteria. After enrolment, participants also completed the PSQI (if enrolled by DASS-42 scoring criteria), DASS-42 (if enrolled by PSQI criteria), Warwick–Edinburgh Mental Wellbeing Scale (WEMWBS), Positive and Negative Affect Schedule (PANAS), and WHO-QoL (quality of life)-BREF questionnaires to provide the status of their emotional distress in the form of mental wellbeing, emotional feelings, and quality of life.

The DASS-42 is a 42-item self-report instrument widely used in the clinical diagnosis and outcome monitoring of three related negative emotional states of depression (D), anxiety (A), and stress (S)/tension (Lovibond and Peter F. L., 1995; Wagar and Amir, 2020). The DASS-42 tool assesses the degree of depression, anxiety, and stress in the form of a total score for each of these conditions, with a higher score denoting a more severe emotional state (Table 1 and Supplementary Figure S1).

**TABLE 1:** Interpreting the DASS score (Lovibond and Peter F. L., 1995).

Emotional distress status	Depression score	Anxiety score	Stress score
Normal	0–9	0–7	0–14
Mild	10–13	8–9	15–19
Moderate	14–20	10–14	19–25
Severe	21–27	15–19	26–33
Extremely severe	28+	20+	34+

The self-report PSQI (Buysse et al., 1989; Hashmi et al., 2014) scale is an effective tool used in the clinical evaluation of sleep disturbances. It measures the quality and patterns of sleep across seven domains: subjective sleep quality, sleep latency, sleep duration, habitual sleep efficiency, sleep disturbances, and use of sleep medication. The component scores are summed to produce a global score (range 0–21). Higher scores indicate worse sleep quality. A score of >5 is considered as significant sleep disturbance (Supplementary Figure S2).

The WEMWBS is a 14-item scale of positively worded statements covering feelings and functioning aspects of mental wellbeing (Tennant et al., 2007; Waqas et al., 2015). The score of WEMWBS is calculated by summing the scores of 14 individual items. The total score ranges from 14 to 70, with higher scores indicating greater positive mental wellbeing (Supplementary Figure S3).

The PANAS (short version) is a widely used tool that measures the affective state using 10 different words that describe feelings and emotions (Watson et al., 1988; Kercher, 1992; Akhter, 2017). The affective state is measured on two scales: one scale measures the positive affective state, and the other measures the negative affective state. Positive affectivity refers to positive emotions and expressions such as joy, cheerfulness, or even contentment, while negative affectivity refers to negative emotions and expressions such as anger, fear, or sadness. The score of each affective state is calculated by summing the scores of individual five items, and ranges from 5 to 25, with a higher positive affectivity score indicating being proactive and enthusiastic, while a higher negative affectivity score shows being disengaged (emotionally detached) (Supplementary Figure S4).

The self-report WHO-QoL-BREF is a 26-item scale designed to measure the impact of disease and impairment on daily activities and behaviour, perceived health, disability, and functional capacity (WHO, 2004; Saqib Lodhi et al., 2017). The scale measures the impact on overall quality of life and general health across four domains: physical health, psychological health, social relationships, and environment. Each domain score is calculated by summing up the component item scores, ranging from 2 to 10 for overall QoL and general health, from 7 to 35 for physical health, from 6 to 30 for psychological health, from 3 to 15 for social relationships, and from 8 to 40 for environment, where higher scores represent a better quality of life.

The participants made two visits in total to the clinic: a screening/baseline visit (V1) and a 3-week follow-up (end of study) visit (V2). An improvement in the DASS-42 questionnaire comprising depression, anxiety, and stress scores or the PSQI questionnaire comprising sleep quality scores was the primary endpoint of this study, while an improvement in the WEMWBS, PANAS, and WHO-QoL-BREF scores was the secondary endpoint of this study.

### 2.4 Statistical analysis

Data were analysed using the Statistical Package for Social Sciences (SPSS; v28.0.1.1), applying standard statistical thresholds (*p* <0.05), which were tested for normality using the Kolmogorov–Smirnov test. Pearson’s correlations were used to assess the associations between the primary outcomes of interest. Independent samples *t*-test, Mann–Whitney U-test, or chi-squared tests were performed to detect the differences between demographic and anthropometric data at baseline. The scores on each questionnaire were entered into separate 2 × 2 repeated measures of ANOVA’s, with the group (supplementation and placebo) as the between-subject factor and time (baseline and follow-up) as the within-subject factor. When assumptions of sphericity were violated, Greenhouse–Geisser corrections were applied.

## 3 Results

### 3.1 Participants’ baseline characteristics and treatment allocation

**FIGURE 1:**
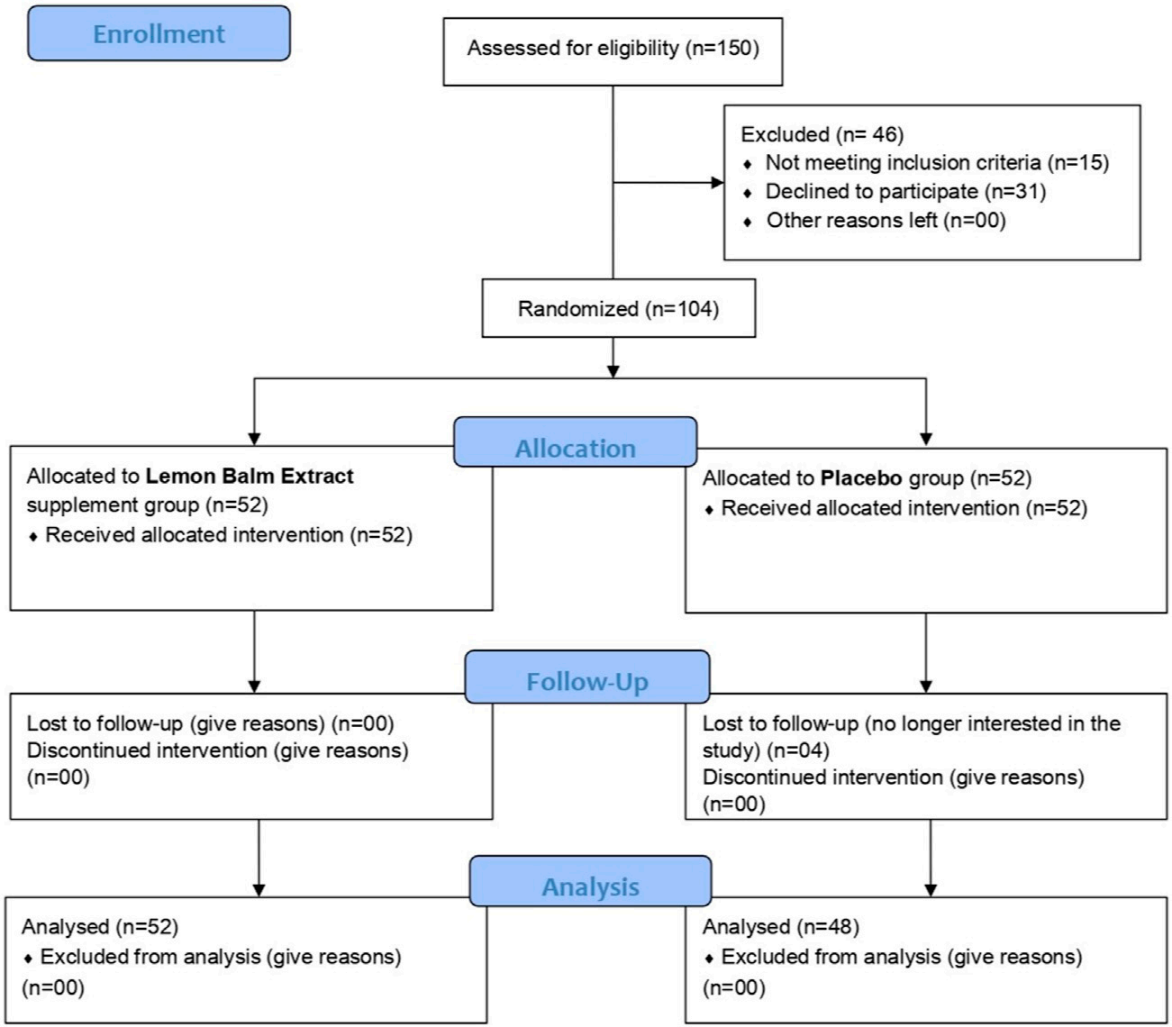
Study CONSORT flow diagram.

Figure 1 shows the flow diagram of the Consolidated Standards of Reporting Trials (CONSORT). In total, 150 participants were initially screened to participate in the study, of which 104 subjects fulfilled the study inclusion/exclusion criteria and were randomly assigned to receive either the MO extract supplement or the placebo using the random number sequence function in Excel at a 1:1 allocation ratio. Randomisation was performed by an independent member of the clinical team who had no role in the study. Out of those randomised participants, 100 participants (supplement group *n* = 52 and placebo group *n* = 48) completed the study and were included in the final analysis. At baseline, participants’ levels of emotional distress and sleep disorder were significantly correlated with each other, i.e., depression–anxiety: *r* (100) = 0.650, *p <*0.001; depression–stress: *r* (100) = 0.758, *p <*0.001; anxiety–stress: *r* (100) = 0.639, *p <*0.001; PSQI–depression: *r* (100) = 0.202, *p =* 0.044; PSQI–anxiety: *r* (100) = 0.316, *p* = 0.001; and PSQI–stress: *r* (100) = 0.175, *p* = 0.081. The demographic characteristics of the participants at baseline are shown in Table 2. Participants’ mean age was 31 ± 9.18 years (ranging 18–60 years), and they were mostly women, (study population were mostly women, i.e., 64.0%). At baseline, both groups were reasonably balanced and did not differ significantly in age, body mass index, marital status, or residence, except for sex, with more men in the supplement group vs. the placebo group (24 vs. 12, *p* = 0.028).

**TABLE 2:** Demographic characteristics of the study subjects.

	Supplement (*n* = 52)	Placebo (*n* = 48)	t/U/χ^2^	*P* value
Sex (men/women), *n*	24/28	12/36	4.85	**0.028**
Age (M ± SD) years	30.96 ± 8.62	31.04 ± 9.84	1221.50	0.855
BMI (M ± SD) kg/m^2^	23.80 ± 4.42	23.89 ± 4.20	−0.104	0.918
Marital status (married/single)	33/19	30/18	0.01	0.921
Residence (urban/rural)	34/18	37/11	1.66	0.198

M, mean; SD, standard deviation; BMI, body mass index. A *p* value of <0.05 is considered statistically significant (denoted in bold).

### 3.2 Supplementation effects on mental health and wellbeing and affective state

**FIGURE 2:**
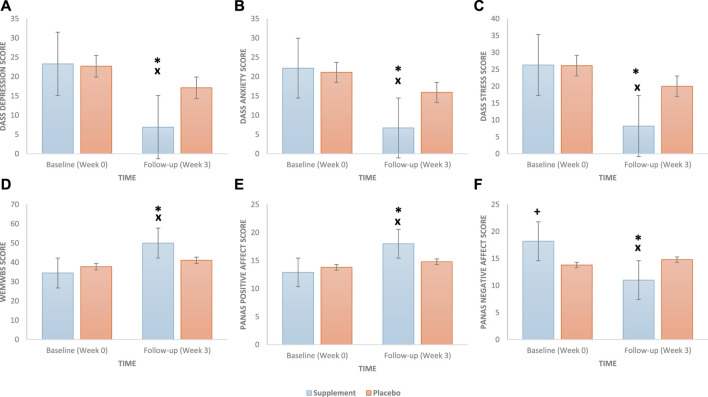
Effect of a daily oral 400 mg phospholipid carrier-based *Melissa officinalis* L. aqueous extract supplementation vs. placebo on study participants’ emotional distress condition scores: **(A)** depression, **(B)** anxiety, **(C)** stress, **(D)** mental wellbeing, **(E)** positive affect, and **(F)** negative affect (bars represent the standard error). DASS, Depression, Anxiety, and Stress Scale; WEMWBS, Warwick–Edinburgh Mental Wellbeing Scale; PANAS, Positive and Negative Affect Schedule. A *p* value of <0.05 is considered statistically significant. +, significant group differences at baseline; x, significant group differences at follow-up; *, significant group × time interaction.

The results are shown in Table 3 and Figure 2. At baseline, no significant differences were observed between the supplement and placebo groups on the DASS-42 Depression (Figure 2A), Anxiety (Figure 2B), and Stress (Figure 2C) scores. At 3-week follow-up, significant group × time interactions were observed in the DASS-42 Depression [F(1, 98) = 57.809, *p* <0.001, η^2^ = 0.371], Anxiety [F(1, 98) = 58.157, *p* <0.001, η^2^ = 0.372], and Stress [F(1, 98) = 52.868, *p* <0.001, η^2^ = 0.350] scores, such that the supplement group showed significant improvements (decrease in the score) in the measures of emotional distress between baseline and follow-up compared to the placebo group. Additionally, there was a statistically significant difference between the two groups (supplement vs. placebo) in the follow-up score in all the measures of emotional distress (all *p* values <0.001). The main effects of time and group are presented in the Supplementary Table S1.

**TABLE 3:** DASS-42, WEMWBS, PANAS, PSQI, and WHO-QoL-BREF questionnaire scores at baseline and follow-up and differences between groups at baseline, follow-up, and group × time interactions.

Measure	Group	*n*	Baseline score (M ± SD)	*p*	Follow-up score (M ± SD)	*p*	(Group × Time) *p*
Mental Health DASS-42							
** **Depression	Supplement	52	23.3 ± 7.9	0.676	6.9 ± 4.5	**<0.001**	**<0.001**
	Placebo	48	22.7 ± 8.1	—	17.1 ± 8.7	—	—
Anxiety	Supplement	52	22.2 ± 7.2	0.498	6.7 ± 3.8	**<0.001**	**<0.001**
	Placebo	48	21.1 ± 8.0	—	15.9 ± 7.3	—	—
Stress	Supplement	52	26.3 ± 7.7	0.860	8.2 ± 4.8	**<0.001**	**<0.001**
	Placebo	48	26.1 ± 6.9	—	20.0 ± 9.4	—	—
WEMWBS	Supplement	52	34.5 ± 9.5	0.070	50.0 ± 9.0	**<0.001**	**<0.001**
	Placebo	48	37.8 ± 8.3	—	41.1 ± 9.8	—	—
PANAS							
** **Positive affect	Supplement	52	12.9 ± 3.2	0.172	18.0 ± 3.0	**<0.001**	**<0.001**
	Placebo	48	13.8 ± 3.0	—	14.8 ± 3.4	—	—
Negative affect	Supplement	52	18.2 ± 3.1	**0.038**	11.0 ± 3.2	**<0.001**	**<0.001**
	Placebo	48	16.5 ± 4.6	—	15.0 ± 4.6	—	—
Sleep quality							
** **PSQI	Supplement	52	10.9 ± 4.3	**0.006**	3.5 ± 2.6	**<0.001**	**<0.001**
	Placebo	48	8.6 ± 3.8	—	6.6 ± 3.3	—	—
Quality of life							
** **QoL total	Supplement	52	5.0 ± 1.3	**0.002**	7.3 ± 1.3	**0.002**	**<0.001**
	Placebo	48	6.0 ± 1.4	—	6.4 ± 1.5	—	—
Physical	Supplement	52	17.9 ± 3.2	0.106	23.5 ± 3.0	**<0.001**	**<0.001**
	Placebo	48	19.0 ± 3.3	—	20.0 ± 3.5	—	—
Psychological	Supplement	52	15.5 ± 4.0	**0.021**	19.3 ± 3.6	**0.004**	**<0.001**
	Placebo	48	17.1 ± 2.7	—	17.4 ± 2.7	—	—
Social	Supplement	52	8.1 ± 2.3	0.082	10.8 ± 2.3	**0.007**	**<0.001**
	Placebo	48	8.9 ± 2.0	—	9.5 ± 2.2	—	—
Environmental	Supplement	52	17.2 ± 5.6	**<0.001**	22.5 ± 8.3	0.710	**<0.001**
	Placebo	48	21.9 ± 6.2	—	22.0 ± 6.2	—	—

M, mean; SD, standard deviation; DASS, Depression, Anxiety, and Stress Scale; PSQI, Pittsburgh Sleep Quality Index; WEMWBS, Warwick–Edinburgh Mental Wellbeing Scale; PANAS, Positive and Negative Affect Schedule; QoL, quality of life.

A *p* value of <0.05 is considered statistically significant (denoted in bold).

At baseline, there were no significant differences between the supplement and placebo groups on the WEMWBS (Figure 2D) and PANAS positive affect (Figure 2E) scores; however, the supplemented group (vs. placebo) reported higher PANAS negative affect (Figure 2F) scores (*p* = 0.038). At the 3-week follow-up, significant group × time interactions were observed for the WEMWBS score [F(1, 98) = 53.617, *p* <0.001, η^2^ = 0.354] and PANAS positive [F(1, 98) = 35.605, *p* <0.001, η^2^ = 0.266] and negative [F(1, 98) = 71.322, *p* <0.001, η^2^ = 0.421] affect scores, such that the supplement group showed significant improvements in the measures of mental wellbeing (increase in the score) and affective state (increase in the score of positive affect and decrease in the score of negative affect) between baseline and follow-up compared to the placebo group. Additionally, there was a statistically significant difference between the two groups (supplement vs. placebo) at the follow-up WEMWBS and PANAS positive and negative affect scores (all *p* values <0.001). The main effects of time and group are presented in the Supplementary Table S1.

### 3.3 Supplementation effects on sleep quality and quality of life

**FIGURE 3:**
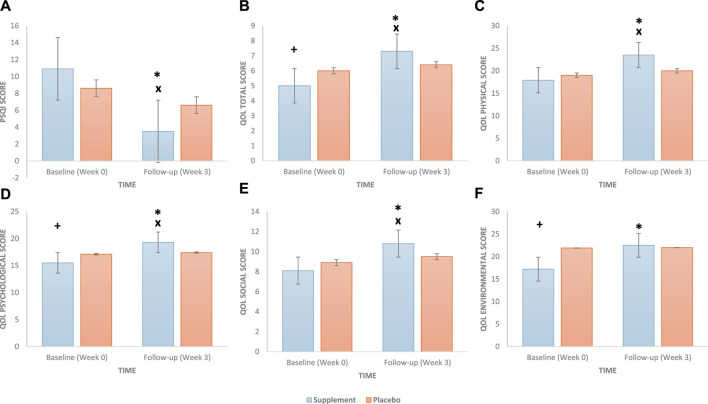
Effect of a daily oral 400 mg phospholipid carrier-based *Melissa officinalis* L. aqueous extract supplementation vs. placebo on study participants: **(A)** sleep quality (PSQI), **(B)** quality-of-life total, **(C)** quality-of-life physical, **(D)** quality-of-life psychological, **(E)** quality-of-life social, and **(F)** quality-of-life environmental scores (bars represent the standard error). PSQI, Pittsburgh Sleep Quality Index. A *p* value of <0.05 is considered statistically significant. +, significant group differences at baseline; x, significant group differences at follow-up; *, significant group × time interaction.

The results are shown in Table 3 and Figure 3. There was a significant difference in the baseline PSQI score (*p* = 0.006) between the two groups, with the supplement group reporting higher PSQI scores (i.e., poor sleep) compared to the placebo group (Figure 3A). At the 3-week follow-up, a significant group × time interaction was found [F(1, 98) = 47.402, *p* <0.001, η^2^ = 0.326], such that the supplement group showed significant improvements (decrease in the score) in sleep quality between baseline and follow-up compared to the placebo group. Additionally, there was a statistically significant difference between the two groups’ (supplement vs. placebo) sleep quality scores at the follow-up (*p* <0.001). The main effects of time and group are presented in the Supplementary Table S1.

Moreover, there were no significant differences between the supplement and placebo groups at baseline for the physical (Figure 3C) and social (Figure 3E) QoL subscales; however, the supplement group (vs. placebo) reported worse total (*p* = 0.002) (Figure 3B), psychological (*p* = 0.021) (Figure 3D), and environmental (*p* <0.001) (Figure 3F) QoL scores. At the 3-week follow-up, significant group × time interactions were observed for total QoL [F(1, 98) = 42.391, *p* <0.001, η^2^ = 0.302], physical [F(1, 98) = 59.673, *p* <0.001, η^2^ = 0.378], psychological [F(1, 98) = 36.524, *p* <0.001, η^2^ = 0.272], social [F(1, 98) = 35.055, *p* <0.001, η^2^ = 0.263], and environmental [F(1, 98) = 42.204, *p* <0.001, η^2^ = 0.301] subscales, such that the supplement group showed significant improvements (increase in score) in all quality-of-life measures between baseline and follow-up compared to the placebo group. Additionally, at follow-up, there was a statistically significant difference between the two groups (supplement vs. placebo) in all QoL measures (all *p* values <0.001) except for the environmental subscale (*p* = 0.710). The main effects of time and group are presented in the Supplementary Table S1.

### 3.4 Compliance, safety, and tolerability of the supplement

Participants’ compliance with the supplement/placebo intake was checked through a weekly phone call. At the completion of the 3-week study period, none of the participants returned any leftover supplement/placebo. Hence, verbal affirmation was taken as a measure of compliance, and most of the participants (>95%) reported adherence to the study protocol. Except a few cases of mild stomach upset, which were equally distributed among the groups (four in the supplement group and five in the placebo group), no specific treatment-emergent effects or serious adverse events were reported by any of the study participants.

## 4 Discussion

In the present study, we investigated the impact of a 3-week-long supplementation of an innovative phospholipid carrier-based formulation of a standardised MO extract (daily 400 mg dosage) on emotional distress and related conditions. Findings revealed that the consumption of MO extract improved depression, anxiety, and stress scores and sleep quality in individuals with a moderate degree of depression, stress, anxiety, or sleep complaints. Additionally, we found that the extract improved overall mental wellbeing, affective state, and quality of life.

One of the notable outcomes of this study is the significant reduction in depression, anxiety, and stress levels in participants who received MO phospholipid extract supplementation. It is noteworthy that these improvements were observed only after 3 weeks of supplementation, suggesting a relatively rapid onset of therapeutic effects of the extract. These findings are broadly in line with the previous research showing the contemporary role of MO extract as a “calming agent” and a mild sedative following acute and chronic supplementation (Kennedy et al., 2002; Kennedy et al., 2003; Kennedy et al., 2004; Cases et al., 2011; Alijaniha et al., 2015; Soltanpour et al., 2019). It is important to note that, unlike our study utilising well-known and clinically validated scales to measure depression, stress, and anxiety in healthy individuals, previous research has measured calmness/anxiety by utilising visual analogue scales in unhealthy individuals (e.g., individuals undergoing coronary artery bypass surgery); hence, our results, in fact, extend the previous findings and show a potential therapeutic impact of the MO extract on subclinical conditions (such as elevated depression, anxiety, and stress levels) in healthy individuals.

Additionally, participants who consumed MO extract experienced a significant increase in overall mental wellbeing and positive affective state and a significant decrease in negative affective state, as evidenced by improvements in the WEMWBS and PANAS scores, respectively. This outcome suggests that in addition to its calming effect, MO phospholipid extract also fosters positive psychological wellbeing and affective state, as well as mitigates negative emotional states. Given that the supplement and placebo groups differed in baseline negative affect scores, the effect of MO supplementation on alleviating negative emotional feelings should be interpreted with caution. Nevertheless, this improvement in emotional states aligns with the observed reductions in the symptoms of depression, anxiety, and stress and underscores the comprehensive impact of MO supplementation on mental health.

In the realm of sleep quality, consistent with the previous research, participants who consumed the MO phospholipid extract demonstrated a significant improvement in the total PSQI score (Cases et al., 2011; Haybar et al., 2018; Heydari et al., 2018; Soltanpour et al., 2019), highlighting its potential as a holistic approach to sleep management. Given that the MO extract positively affected mental health and wellbeing, it could also indirectly affect the quality of sleep by reducing mental health symptomatology. However, similar to negative affect, participants in the supplement and placebo groups differed in sleep quality at the baseline, hence, the results should be interpreted with caution.

Another important aspect of this study is that the MO supplement elicited a positive impact on various domains of QoL, e.g., physical and social aspects. Although there were differences between the supplement and placebo groups in terms of baseline scores of the total QoL, psychological, and environmental subscales, which may have driven the observed effects, the treatment group exhibited improvements in all quality-of-life measures at 3 weeks follow-up. These improvements suggest that MO supplementation has the potential to enhance overall life satisfaction and functioning and further add to the evidence base.

It is important to highlight that none of the participants who received the supplement (daily dosage 400 mg) reported any serious side effects, such as those associated with conventional antidepressants and anxiolytic drugs, at least during the 3-week study period, demonstrating the excellent safety and tolerability of the phospholipid carrier-based formulation of MO extract supplementation. These results are consistent with the previously reported studies with other MO formulations (Ballard et al., 2002; Akhondzadeh et al., 2003; Burns et al., 2011; Cases et al., 2011; Alijaniha et al., 2015; Noguchi-Shinohara et al., 2015; Soltanpour et al., 2019; Araj-Khodaei et al., 2020). In general, MO extract supplementation is considered a safe alternative therapy for the management of emotional distress and related conditions. Noguchi-Shinohara et al. (2020) analysed that in patients with mild dementia due to Alzheimer’s disease (AD), a daily dose of 500 mg of MO extract supplementation for 24 weeks failed to produce any adverse events. Asadi et al. (2019) observed that in patients with type 2 diabetes, a daily dose of 700 mg of MO extract supplementation for 3 months was safe and tolerable. However, it has been repeatedly pointed out that MO should be used with caution in patients with thyroid dysfunction as it mediates thyroid hormone inhibition (Ulbricht et al., 2005). Overall, no common serious side effects have so far been reported for MO extract supplementation (Rafieian-Kopaei et al., 2013) in otherwise healthy adults or when used in nutritional amounts. As a matter of fact, in 2013, the European Medicines Agency approved MO leaf extract prepared as a herbal infusion, dry extract, or fluid extract as a non-prescription medicine for alleviating mild symptoms of mental stress and fostering sleep, and for relieving mild digestive disorders, which included bloating and flatulence (HMPC Community herbal monograph on Melissa *officinalis* L. folium, 2007). The European Scientific Cooperative on Phytotherapy (ESCOP) also recommends the use of the MO extract for tenseness, restlessness, and irritability (Escop European Scientific Cooperative on Phytotherapy, 2003). In the US, the MO extract has received FDA GRAS (Generally Recognised as Safe) status for use as a food ingredient (US Food and Drug Administration, 2016).

Due to the demonstrated neuropharmacological therapeutic effects of the oral MO extract, as observed in the present study, it is currently one of the most popular over-the-counter supplements for the management of low mood conditions, particularly in individuals who are refractory to conventional antidepressant/anxiolytic drugs. Among the reported clinical trials, the MO extract supplement dosage of 300 to 1,600 mg has shown benefits in the management of depression, anxiety, stress, and poor sleep. In terms of duration, studies have shown that a period between 10 days and 8 weeks is required to improve depressive symptoms. Some clinical trials have shown that the shortest duration of MO oral supplementation that led to improvement in anxiety disorders was 5–7 days (Soltanpour et al., 2019; Saeidi et al., 2020). In the presence of available evidence on scientific rationale, clinical efficacy, and safety, we propose that phospholipid carrier-based MO extract supplementation may serve as a promising short-term natural intervention for individuals seeking relief from symptoms of depression, anxiety, and stress while simultaneously enhancing their overall mental wellbeing.

With regards to possible mechanisms of action, the beneficial effects of MO supplementation on low mood and/or anxiety are believed to be largely driven by the natural GABAergic properties of the supplement. γ-Amino butyric acid is the principal inhibitory neurotransmitter in the central nervous system (Brambilla et al., 2003; Granger et al., 2005) that has been implicated in a range of behaviours, including, but not limited to, low mood, anxiety, stress regulation, memory enhancement, and circadian rhythm (Hepsomali et al., 2020). Brain cells low GABA levels and/or impaired GABA functioning are known to be associated with the aetiology and maintenance of acute and chronic stress (Jie et al., 2018), anxiety, depressive disorders (Nemeroff, 2003), and insomnia (Gottesmann, 2002). *Melissa officinalis* L. inhibits GABA-T (Awad et al., 2007; Awad et al., 2009; Yoo et al., 2011), resulting in an increase in brain GABA levels and a subsequent increase in GABA transmission. Amongst the bioactive chemical constituents of the MO extract, rosmarinic acid (a hydroxycinnamic acid ester polyphenol) is the major ingredient and an important biomarker used for the standardisation of MO extract (Arceusz et al., 2015; Noguchi-Shinohara et al., 2015; Shakeri et al., 2016). Rosmarinic acid has the ability to cross the blood–brain barrier (Falé et al., 2011). Therefore, the psychopharmacological effects of the MO extract are predominantly attributed to RA (Awad et al., 2007; Awad et al., 2009; Jin et al., 2013; Sahin et al., 2016; Ghazizadeh et al., 2020), possibly acting in a synergistic or additive way with other biochemical mechanisms (Savelev et al., 2003), such as GABA-T inhibition by ursolic and oleanolic acid contents present in the MO extract (Awad et al., 2009; Ibarra et al., 2010). The *Melissa officinalis* L. extract has also shown an affinity for GABA_A_ receptors (Salah and Jäger, 2005; Abuhamdah et al., 2008; Sahin et al., 2016), leading to an enhancement in GABAergic signalling. GABA receptors are targets for many pharmacological treatments for insomnia, such as benzodiazepines. GABA_A_ receptor modulation is one of the four key mechanisms of action of the approved pharmacological therapies for insomnia (Avidan and Neubauer, 2017).

The phospholipid carrier-based (Phytosome™) MO extract (Relissa™) used in the present study is an innovative food-grade formulation developed to achieve increased bioavailability and therapeutic effects of the extract. The Phytosome™ carrier is a solid botanical dispersion recently reported to improve the effectiveness and target reach of a wide range of natural compounds, such as quercetin (Riva et al., 2019b), berberine (Rondanelli et al., 2021), curcumin (Pivari et al., 2022), boswellic acids (Riva et al., 2019a), and other botanical extracts, without safety concerns. We can hypothesise that the possible “calming effect” observed in the present study after 3-week supplementation of the phospholipid carrier-based MO extract could also be due to this new formulation used; however, more studies are needed to better clarify the role of the Phytosome™ carrier.

However, it is essential to acknowledge some limitations of this study, such as evaluation of only one dose of MO extract, lack of compliance measurements, and the absence of evaluation of therapeutic effect in the form of objective biomarkers such as cortisol, C-reactive protein (CRP), adrenocorticotropic hormone (ACTH), dehydroepiandrosterone (DHEA), and corticosterone levels etc, which should be considered in future studies. In the current study, although the men/women ratio was somewhat balanced in the supplement group, there were more female participants in the placebo group. Given that the NIH Policy (Arnegard et al., 2020) recommends considering sex as a biological variable, future studies should consider this crucial factor in the recruitment and analysis stages. Although this study primarily focused on short-term effects (3 weeks of supplementation) on mental health and related conditions, it is essential to acknowledge the potential for sustained benefits over extended periods. Future research should explore the long-term impact of MO phospholipid extract supplementation on mental health, wellbeing, and sleep quality to ascertain its utility as a continuous intervention. While this study has demonstrated the positive effects of MO supplementation, the underlying mechanisms remain a subject for further investigation. Understanding how MO’s bioactive compounds interact with neuronal pathways and physiological processes could shed light on its therapeutic actions and guide the development of more targeted interventions. Additionally, research studies should explore optimal dosage regimens and potential interactions with other interventions or medications. The results observed in the present study underscore the potential breadth and depth of MO phospholipid extract supplementation’s effects on mental health and related conditions. They hint at the possibility of enduring benefits, gender-specific responses, and opportunities for elucidating the mechanisms that drive these improvements. As such, they emphasise the need for future research to explore these facets in greater detail and promote a more comprehensive understanding of MO’s extract therapeutic potential.

## 5 Conclusion

In conclusion, this rigorous, randomised, prospective, multi-centre, double-blinded, placebo-controlled clinical study has provided compelling evidence of the beneficial effects of MO phospholipid extract supplementation on mental health, wellbeing, and sleep quality. The findings of this study have several important implications and open avenues for future research and clinical applications.

## Data Availability

The original contributions presented in the study are included in the article/Supplementary Material; further inquiries can be directed to the corresponding author.
