# Supplementary material for: The possible “calming effect” of subchronic supplementation of a standardised phospholipid carrier-based Melissa officinalis L. extract in healthy adults with emotional distress and poor sleep conditions: results from a prospective, randomised, double-blinded, placebo-controlled clinical trial
Source: Front Pharmacol. 2023 Oct 19;14:1250560. doi: 10.3389/fphar.2023.1250560 (PMC10620697; doi:10.3389/fphar.2023.1250560)
Supplement: Supplementary file 1 [file Image5.PDF]

WHOQOL-BREF

ہدایات:

اس سوالنامہ میں آپ کی زندگی کے معیار ، صحت اور زندگی کے دیگر پہلوؤں کے بار ے میں پوچھا جائے گا۔ براہ مہربانی اپ تمام سوالات کے جواب دیں۔ اگر آپ کسی سوال کے جواب کے بارے میں یقینی طور پر کچھ نہیں کہ سکتے تو سب سے مناسب جواب کا چناؤ کریں ۔ عموماً یہ وہ جواب ہو سکتا ہے جو کہ آپ کے ذہن میں سب سے پہلے آئے۔ آپ سے گزارش ہے کہ اپنے ذاتی معیار ، اُمیدیں ، خوشیاں اور خدشات ذہن میں رکھیں۔ سوالات دیتے وقت پچھلے دو ہفتوں کی زندگی کو ذہن میں رکھیں۔

آپ سے گزارش ہے کہ ہر سوال کو غور سے پڑھیں اور اپنے احساسات کاجائزہ لیں اور پھر اُس نمبر پر دائرہ لگائیں جو آپ کے احساسات کو بہتر طور پر ظاہر کرتا ہو۔

بہت اچھا<br>5	اچھا<br>4	نہ اچھا نہ برا<br>3	برا<br>2	بہت برا<br>1	آپ اپنے معیار کی زندگی کو کس درجہ کامحسوس کرتے ہیں۔	1
بہت مطمئن<br>5	مطمئن<br>4	نہ مطمئن نہ غیر مطمئن<br>3	غیر مطمئن<br>2	بہت غیر مطمئن<br>1	آپ اپنی صحت سے کس حد تک مطمئن ہیں۔	2

مندرجہ ذیل سوالات میں آپ کچھ مخصوص چیزوں کے بارے میں پوچھا جائے گا کہ ان سے آپ کا پچھلے دو ہفتوں میں کس حد تک تجربہ ہو ا ہے۔

بہت ہی زیادہ	بہت زیادہ	درمیانی حد تک	تھوڑابہت	باکل نہیں	آپ کس حد تک محسوس کرتے ہیں کہ جسمانی درد آپ کے لئے وہ کام کرنے میں رکاوٹ بنتی ہے جس کا کرنا آپ کے لئے ضروری ہوتا ہے۔	3
5	4	3	2	1	روزمرہ کاموں کی ادائیگی کے لئے آپ کس حد تک طبی علاج کی ضرورت پڑتی ہے۔	4
5	4	3	2	1	آپ کس حد تک اپنی زندگی سے لطف اندوز ہوتے ہیں۔	5
5	4	3	2	1	آپ کس حد تک اپنی زندگی کو بامعنی محسوس کرتے ہیں۔	6
5	4	3	2	1	آپ کس حد تک اپنے آپ کو توجہ مرکوز کرنے کے قابل سمجھتے ہیں۔	7
5	4	3	2	1	آپ روزمرہ زندگی میں اپنے آپ کو کس حد تک محفوظ کرتے ہیں۔	8
5	4	3	2	1	آپ کے ارد گرد کا طبعی ماحول کس حد تک صحت مندانہ ہے۔	9
5	4	3	2	1	کیا آپ روزمرہ زندگی کے لئے مناسب توانائی محسوس کرتے ہیں۔	10
5	4	3	2	1	کیا آپ کے لئے اپنی ظاہری جسمانی شکل وصورت قابل قبول ہے۔	11
5	4	3	2	1	کیا آپ کے پاس اپنی ضروریات پوری کرنے کے لئے مناسب پیشہ موجود ہے۔	12
5	4	3	2	1	آپ کو روزمرہ زندگی گزارنے سے متعلق کتنی ضروری معلومات دستیاب ہیں۔	13
5	4	3	2	1	آپ کو سیر وتفریح کے مواقع کس حد تک میسر ہیں۔	14
5	4	3	2	1	آپ اپنے ارد گرد جسمانی طور پر کس حد تک چلنے پھرنے کے قابل ہیں۔	15

مندرجہ ذیل سوالات میں آپ سے پوچھا گیا ہے کہ پچھلے دو ہفتوں سے آپ نے اپنے زندگی کے مختلف پہلوؤں کے حوالے سے کس قدر اچھا یا مطمئن محسوس کیا۔

16	آپ اپنی نیند سے کس حد تک مطمئن ہیں	انتہائی غیر مطمئن 1	غیر مطمئن 2	نہ مطمئن نہ غیر مطمئن 3	مطمئن 4	انتہائی مطمئن 5
17	آپ اپنی روزمرہ کام سرانجام دینے کی صلاحیت سے کس حد تک مطمئن ہیں۔	1	2	3	4	5
18	آپ اپنی کام کرنے کی صلاحیت سے کس حد تک مطمئن ہیں۔	1	2	3	4	5
19	آپ اپنی ذات سے کس حد تک مطمئن ہیں۔	1	2	3	4	5
20	آپ اپنے تعلقات سے کس حد تک مطمئن ہیں۔	1	2	3	4	5
21	آپ اپنی جنسی زندگی سے کس حد تک مطمئن ہیں۔	1	2	3	4	5
22	آپ اپنے دوستوں سے ملنے والی مدد سے کس حدتک مطمئن ہیں۔	1	2	3	4	5
23	آپ اپنی رہائش کی جگہ کے حالات سے کس حد تک مطمئن ہیں۔	1	2	3	4	5
24	آپ طبعی سہولتوں تک اپنی رسائی سے کس حد تک مطمئن ہیں۔	1	2	3	4	5
25	آپ اپنے ذرائع آمدورفت سے کس حد تک مطمئن ہیں۔	1	2	3	4	5
26	آپ کس حد تک منفی احساسات کا شکار رہتے ہیں مثلاً اداسی، مایوسی ، پریشانی اور افسردگی وغیرہ۔	کبھی نہیں 1	بعض اوقات 2	کبھی کبھار 3	بہت زیادہ 4	ہمیشہ 5

Overall Quality of Life and General Health score: (Sum of Q1, 2) = ……………………………

Domain 1 (Physical Health) score: (Sum of Q 3, 4, 10, 15, 16, 17, 18) = ……………………………

Domain 2 (Psychological Health) score: (Sum of Q 5, 6, 7, 11, 19, 26) = ……………………………

Domain 3 (Social relationships) score: (Sum of Q 20, 21, 22) = ……………………………

Domain 4 (Environment) score: (Sum of Q 8, 9, 12, 13, 14, 23, 24, 25) = ……………………………

WHOQOL-BREF Score Interpretation

The WHOQOL-BREF addresses four quality of life domains: physical health, psychological health, social relationships, and environment. Domain scores are scaled in a positive direction (i.e., higher scores denote higher quality of life).

The WHOQOL-BREF total score is presented between 26 and 156, where higher scores represent higher levels of quality of life.

Overall Quality of Life and General Health: Score range, 2 – 10.

Domain 1: Score range, 7 – 35.

Domain 2: Score range, 6 – 30.

Domain 3: Score range, 3 – 15.

Domain 4: Score range, 8 – 40.
