# Supplementary material for: The possible “calming effect” of subchronic supplementation of a standardised phospholipid carrier-based Melissa officinalis L. extract in healthy adults with emotional distress and poor sleep conditions: results from a prospective, randomised, double-blinded, placebo-controlled clinical trial
Source: Front Pharmacol. 2023 Oct 19;14:1250560. doi: 10.3389/fphar.2023.1250560 (PMC10620697; doi:10.3389/fphar.2023.1250560)
Supplement: Supplementary file 2 [file Table1.DOCX]

|  |  | ***F*** | ***df*** | ***p*** | ***η^2^*** |
| --- | --- | --- | --- | --- | --- |
| **DASS Depression** | Time | 235.966 | 1,98 | <.001 | .707 |
|  | Group | 12.934 | 1,98 | <.001 | .117 |
| **DASS Anxiety** | Time | 237.247 | 1,98 | <.001 | .708 |
|  | Group | 11.777 | 1,98 | <.001 | .107 |
| **DASS Stress** | Time | 212.399 | 1,98 | <.001 | .684 |
|  | Group | 22.147 | 1,98 | <.001 | .184 |
| **WEMWBS** | Time | 125.613 | 1,98 | <.001 | .562 |
|  | Group | 2.949 | 1,98 | .089 | .029 |
| **PANAS Positive Affect** | Time | 80.988 | 1,98 | <.001 | .452 |
|  | Group | 4.349 | 1,98 | .040 | .042 |
| **PANAS Negative Affect** | Time | 167.941 | 1,98 | <.001 | .631 |
|  | Group | 2.681 | 1,98 | .105 | .027 |
| **PSQI** | Time | 138.832 | 1,98 | <.001 | .586 |
|  | Group | 0.453 | 1,98 | .503 | .005 |
| **QoL Total** | Time | 93.200 | 1,98 | <.001 | .487 |
|  | Group | 0.000 | 1,98 | .992 | .000 |
| **QoL Physical** | Time | 123.219 | 1,98 | <.001 | .557 |
|  | Group | 4.231 | 1,98 | .042 | .041 |
| **QoL Psychological** | Time | 48.498 | 1,98 | <.001 | .331 |
|  | Group | 0.061 | 1,98 | .806 | .001 |
| **QoL Social** | Time | 90.904 | 1,98 | <.001 | .481 |
|  | Group | 0.388 | 1,98 | .535 | .004 |
| **QoL Environmental** | Time | 44.232 | 1,98 | <.001 | .311 |
|  | Group | 2.618 | 1,98 | .109 | .026 |

**Supplementary Materials**

**Supplementary Table 1.** ANOVA time and group main effects for all outcome measures.

DASS: The Depression, Anxiety, Stress Scale; WEMWBS: The Warwick Edinburgh Mental Wellbeing Scale; PANAS: The Positive and Negative Affect Schedule; PSQI: The Pittsburgh Sleep Quality Index; QoL: Quality of Life.
