# Supplementary material for: The possible “calming effect” of subchronic supplementation of a standardised phospholipid carrier-based Melissa officinalis L. extract in healthy adults with emotional distress and poor sleep conditions: results from a prospective, randomised, double-blinded, placebo-controlled clinical trial
Source: Front Pharmacol. 2023 Oct 19;14:1250560. doi: 10.3389/fphar.2023.1250560 (PMC10620697; doi:10.3389/fphar.2023.1250560)
Supplement: Supplementary file 3 [file Image4.PDF]

Status of Mode

Appendix: Urdu version of short version of positive affect and negative affect scale (PANAS).

لوگ اپنی زندگی میں مختلف قسم کے جذبات محسوس کرتے ہیں۔ آپ مندرجہ ذیل جذبات کس حد تک محسوس کرتے ہیں۔ برائے مہربانی ہر سوال کے لیے صرف ایک جواب کی نشاندہی کیجیے جو آپ کے جذبات کی صحیح عکاسی کرتا ہو۔

بہت یادہ	کسی حد تک	درمیانہ	بہت کم	کبھی نہیں	بیانات	نمبر شمار
5	4	3	2	1	پریشان حال۔	-1
5	4	3	2	1	ڈرا ہوا۔	-2
5	4	3	2	1	پُر جوش۔	-3
5	4	3	2	1	چوکنا/ہوشیار۔	-4
5	4	3	2	1	متاثر کن۔	-5
5	4	3	2	1	تکلیف دہ حالت میں۔	-6
5	4	3	2	1	بے چین/مضطرب۔	-7
5	4	3	2	1	پُر عزم۔	-8
5	4	3	2	1	خوفزدہ۔	-9
5	4	3	2	1	جوشیلا۔	-10

Negative Feeligs (emotions)

Q1 + Q2 + Q6 + Q7 + Q9

Total Positive score =

Positive Feeligs

Q3 + Q4 + Q5 + Q8 + Q10

Total Negative Score =

Score Interpretation

A high positive affectivity score indicates positive feelings that lead to being proactive and enthusiastic. A low positive affect score represents lethargy (lack of energy and enthusiasm) and sadness. A high negative affectivity score highlights negative emotions that lead to a state of distress or being disengaged (emotionally detached). A low negative affect score reflects a state of being calm (relax).
