# Supplementary material for: The possible “calming effect” of subchronic supplementation of a standardised phospholipid carrier-based Melissa officinalis L. extract in healthy adults with emotional distress and poor sleep conditions: results from a prospective, randomised, double-blinded, placebo-controlled clinical trial
Source: Front Pharmacol. 2023 Oct 19;14:1250560. doi: 10.3389/fphar.2023.1250560 (PMC10620697; doi:10.3389/fphar.2023.1250560)
Supplement: Supplementary file 4 [file Image2.PDF]

Pittsburgh Sleep Quality Index (PSQI)

پٹسبرگ نیند کے معیار کا اشاریہ

The Pittsburgh Sleep Quality Index (PSQI) contains 19 self-rated questions.

The 19 self-rated items are combined to form seven "component" scores, each of which has a range of 0-3 points. In all cases, a score of "0" indicates no difficulty, while a score of "3" indicates severe difficulty. The seven component scores are then added to yield one "global" score, with a range of 0-21 points, "0" indicating no difficulty and "21 " indicating severe difficulties in all areas.

ہدایات:

مندرجہ ذیل سوالات پچھلے ایک مہینے میں آپ کی روزمرہ نیند کی عادات کے بارے میں ہیں۔ آپ کے جوابات گزشتہ مہینے میں زیادہ تر دن اور رات کی درست کیفیت کے بارے میں ہونے چاہیں۔ برائے مہربانی سب سوالوں کے جواب دیجئے۔

گزشتہ مہینے میں:

1- آپ کس وقت سونے کے لئے لیٹے؟

Time: ....

2- کتنی دیر میں (منٹوں میں) آپ کو نیند آئی؟

Response	Score
≤15 minutes	0
16-30 minutes	1
31-60 minutes	2
> 60 minutes	3

3- آپ عموماً " صبح کتنے بجے اُٹھتے ہیں؟

Time: ....

4- آپ کی رات کی کُل نیند کتنی ہے؟ (یہ وقت بستر میں لیٹے ہوئے گھنٹوں سے مختلف ہوسکتا ہے)

Response	score
> 7 hours	0
6-7 hours	1
5-6 hours	2
< 5 hours	3

SCORE: 3 2 1 0

ہفتے میں تین دفعہ سے زیادہ	ایک ہفتے میں ایک یا دو دفعہ	ایک ہفتے میں ایک دفعہ سے کم	پچھلے مہینے میں کبھی نہیں	5۔پچھلے ایک ماہ میں آپ کو کتنی بار سونے میں دشواری ہوئی کیونکہ۔۔۔۔
				a۔30منٹ کے اندر نیند نہیں آئی
				b۔رات کے درمیان میں یا صبح بہت جلدی آنکھ کھل گئی۔
				c۔غسل خانے جانے کے لیئے اٹھنا پڑا۔
				d۔سانس لینے میں دُشواری ہورہی تھی۔
				e۔کھانسی یا اُونچے خراٹے آئے۔
				f۔سردی زیادہ لگی۔
				g۔گرمی زیادہ لگی۔
				h۔بُرے خواب آئے۔
				i۔درد محسوس ہوا۔
				j۔کوئی اور وجہ، اس کی وضاحت کیجئے اور اس وجہ سے کتنی بار نیند میں دشواری ہوئی؟
بہت بُرا	بُرا	اچھا	بہت اچھا	6۔پچھلے ماہ میں آپ کی نیند کا مجموعی معیار کیسا تھا؟
				7۔پچھلے ماہ میں آپ نے نیند کے لۓ کتنی مرتبہ دوائیاں استعمال کیں؟ (ڈاکٹر کے نسخے کے ساتھ یا نسخے کے بغیر)
				8۔پچھلے ایک ماہ میں کتنی مرتبہ آپ کو گاڑی (یا موٹر سائیکل) چلاتے ہوئے، کھانا کھاتے ہوئے یا لوگوں سے ملتے جلتے ہوئے مُشکل پیش آئی۔
				9۔پچھلے ایک ماہ میں کوئی بھی کام لگن کے ساتھ کرنے میں کتنی مُشکل پیش آئی؟

PSQI global score calculation

Component 1 score<br>*(Subjective sleep quality)*	**Enter Q6 score here =** ……………………………….
Component 2 score<br>*(Sleep latency)*	Q2 score + Q5a score = ……………………….<br>**Sum of Q2 and Q5a scores** — **Component score**<br>0 — 0<br>1 – 2 — 1 (tick the component score)<br>3 – 4 — 2<br>5 – 6 — 3
Component 3 score<br>*(Sleep duration)*	**Enter Q4 score here =** …………………………………..
Component 4 score<br>*(Habitual sleep efficiency)*	(a) Numbers of hours slept, from Q4 = …………………hours<br>(b) Getting up time, from Q3 = …………………………..<br>(c) Bedtime from Q1 = …………………………<br>(d) Calculate the number of hours spend in the bed = …………………..<br>Habitual sleep efficiency = (a) ÷ (d) × 100 = ………………………….% **Habitual sleep efficiency (%)** — **Component score**<br>> 85% — 0<br>75 – 84% — 1 (tick the component score)<br>65 – 74% — 2<br>< 65% — 3
Component 5 score<br>*(Sleep disturbances)*	Sum of Q5b to Q5j score = ………………………..<br>**Sum of Q5b to Q5j score** — **Component score**<br>0 — 0<br>1 – 9 — 1 (tick the component score)<br>10 – 18 — 2<br>19 – 27 — 3
Component 6 score<br>*(Use of sleep medication)*	**Enter Q7 score here =** ……………………………….
Component 7 score<br>*(Daytime dysfunction)*	Q8 score + Q9 score = …………………………….<br>**Sum of Q8 and Q9 scores** — **Component score**<br>0 — 0<br>1 – 2 — 1 (tick the component score)<br>3 – 4 — 2<br>5 – 6 — 3
PSQI global score	**Sum of components 1-7 scores =** ……………………………
