# Supplementary material for: The possible “calming effect” of subchronic supplementation of a standardised phospholipid carrier-based Melissa officinalis L. extract in healthy adults with emotional distress and poor sleep conditions: results from a prospective, randomised, double-blinded, placebo-controlled clinical trial
Source: Front Pharmacol. 2023 Oct 19;14:1250560. doi: 10.3389/fphar.2023.1250560 (PMC10620697; doi:10.3389/fphar.2023.1250560)
Supplement: Supplementary file 5 [file Image3.PDF]

# وارِوک ۔ ایڈنبرا مینٹل ویلبنگ اِسکیل

## (WEMWBS)

ذیل میں خیالات اور احساسات کے بارے میں کچھ بیانات درج ہیں۔

برائے مہربانی ہر اُس جواب کو منتخب کریں جو آپ کے گذشتہ دو ہفتوں کے تجربے کو بہتر طریقے سے بیان کرتا ہے۔

| ہر وقت | اکثر و بیشتر | بعض اوقات | بہت کم | کبھی نہیں | | |
|---|---|---|---|---|---|---|
| 5 | 4 | 3 | 2 | 1 | مجھے اپنا مستقبل روشن ہونے کا احساس رہا ہے | 1 |
| 5 | 4 | 3 | 2 | 1 | مجھے دوسروں کے لیے کارآمد ہونے کا احساس رہا ہے | 2 |
| 5 | 4 | 3 | 2 | 1 | میں پرسکون محسوس کرتا رہا/رہی ہوں | 3 |
| 5 | 4 | 3 | 2 | 1 | مجھے دوسرے لوگوں میں دلچسپی رہی ہے | 4 |
| 5 | 4 | 3 | 2 | 1 | مجھے اپنے اندر توانائی کا احساس رہا ہے | 5 |
| 5 | 4 | 3 | 2 | 1 | میں مسائل کو اچھی طرح سے حل کرتا رہا/رہی ہوں | 6 |
| 5 | 4 | 3 | 2 | 1 | میری سوچ واضح رہی ہے | 7 |
| 5 | 4 | 3 | 2 | 1 | مجھے اپنے متعلق اچھا محسوس ہوتا رہا ہے | 8 |
| 5 | 4 | 3 | 2 | 1 | مجھے دوسرے لوگوں کے ساتھ قربت کا احساس رہا ہے | 9 |
| 5 | 4 | 3 | 2 | 1 | میں بااعتماد محسوس کرتا رہا/رہی ہوں | 10 |
| 5 | 4 | 3 | 2 | 1 | میں اپنے فیصلے خود کرنے کے قابل رہا/رہی ہوں | 11 |
| 5 | 4 | 3 | 2 | 1 | مجھے یہ احساس رہا ہے کہ لوگ مجھے پیار کرتے ہیں | 12 |
| 5 | 4 | 3 | 2 | 1 | مجھے نئی سرگرمیوں میں دلچسپی رہی ہے | 13 |
| 5 | 4 | 3 | 2 | 1 | میرے اندر مسرت کا جذبہ موجود رہا ہے | 14 |

Total Score (sum of scores of item 1 -14) = ____________________

---

**WEMWBS Scoring**

The WEMWBS total score is obtained by summing the score for each of the 14 items. The latter ranges from 1 – 5 and the total score from 14 – 70.

0 – 32 points: Your mental wellbeing score is very low.

32 – 40 points: Your mental wellbeing score is below average.

40 – 59 points: Your mental wellbeing score is average.

59 – 70 points: Your mental wellbeing score is above average.

Most people have a score between 41 and 59.
