# Supplementary material for: The possible “calming effect” of subchronic supplementation of a standardised phospholipid carrier-based Melissa officinalis L. extract in healthy adults with emotional distress and poor sleep conditions: results from a prospective, randomised, double-blinded, placebo-controlled clinical trial
Source: Front Pharmacol. 2023 Oct 19;14:1250560. doi: 10.3389/fphar.2023.1250560 (PMC10620697; doi:10.3389/fphar.2023.1250560)
Supplement: Supplementary file 6 [file Image1.PDF]

Depression Anxiety Stress Scale (Urdu Version)

براہ مہربانی ہر فقرے کا مطالعہ کریں اور ایک نمبر 2،1،0 یا 3 پر دائرہ لگائیں جو یہ ظاہر کرے کہ گزشتہ ہفتے یہ بیان آپ پر کس حد تک لاگو ہوا کوئی غلط یا درست جوابات نہیں ہیں۔ کسی بھی بیان پر بہت زیادہ وقت صرف مت کریں۔

نمبر شمار	فقرے	مجھ پر بالکل بھی لاگو نہیں ہوتا	مجھ پر کسی حد تک، یا کچھ وقت کے لئے لاگو ہوتا ہے	مجھ پر کافی حد تک، یا کافی وقت کے لئے لاگو ہوتا ہے	مجھ پر بہت حد تک یا زیادہ تر وقت کے لئے لاگو ہوتا ہے
1	میں نے اپنے آپ کو معمولی باتوں کی وجہ سے پریشان پایا	0	1	2	3
2	میں اپنا منہ خشک ہونے کے بارے میں جانتا تھا	0	1	2	3
3	میں کسی بھی قسم کے مثبت احساس نہیں رکھ سکا	0	1	2	3
4	مجھے سانس لینے میں دشواری کا سامنا ہوا (مثلاً سانس کا زیادہ تیزی سے چلنا، جسمانی مشقت کی غیر موجودگی میں سانس لینے میں دقت ہونا)	0	1	2	3
5	میں خود کو کام کرنے کے لیے مستعد نہ پا سکا	0	1	2	3
6	میرا ردعمل صورت حال کی مناسبت سے شدید ہوتا۔	0	1	2	3
7	مجھے لڑکھڑانے/کانپنے کا احساس ہوا (مثلاً ٹانگوں کا جواب دینا)	0	1	2	3
8	مجھے پرسکون رہنا مشکل محسوس ہوا	0	1	2	3

3	2	1	0	میں نے خود کو ایسی صورتحال میں پایا جس نے مجھے بہت پریشان کر دیا۔ میں نے ان کے ختم ہونے پر بہت بہتر محسوس کیا	9
3	2	1	0	مجھے محسوس ہوا کہ میرے پاس آگے کرنے کے لیے کچھ نہیں ہے۔	10
3	2	1	0	میں نے محسوس کیا کہ میں جلدی پریشان ہو جاتا ہوں	11
3	2	1	0	میں نے محسوس کیا کہ میں بہت زیادہ اعصابی توانائی استعمال کر رہا ہوں	12
3	2	1	0	میں نے خود کو غمگین اور افسردہ محسوس کیا	13
3	2	1	0	جب بھی مجھے کسی معاملے میں دیر ہوئی میں نے خود کو بے صبر محسوس کیا (مثلاً لفٹ میں، ٹریفک لائٹس کی وجہ سے یا، انتظار کروانے پر)	14
3	2	1	0	مجھے بے ہوشی کا احساس ہوا	15
3	2	1	0	مجھے احساس ہوا کہ میں نے ہر چیز میں دلچسپی کھو دی ہے	16
3	2	1	0	مجھے احساس ہوا کہ بحیثیت انسان میری کوئی اہمیت نہیں	17
3	2	1	0	مجھے احساس ہوا کہ میں ذرا حساس طبیعت کا مالک ہوں	18
3	2	1	0	زیادہ درجہ حرارت یا جسمانی مشقت کے بغیر بھی مجھے واضح طور پر پسینہ آیا (مثلاً ہاتھوں میں پسینہ آنا)۔	19

3	2	1	0	میں نے بغیر کسی مناسب وجہ کے خوف محسوس کیا	20
3	2	1	0	مجھے احساس ہوا کہ زندگی بڑی بے وقعت ہے	21
3	2	1	0	مجھے کام ختم کرنا مشکل محسوس ہوا	22
3	2	1	0	مجھے نگلنے میں دشواری کا سامنا ہوا	23
3	2	1	0	مجھے اپنے کیے ہوئے کاموں سے کسی لطف کا احساس نہیں ہوا	24
3	2	1	0	کسی بھی جسمانی مشقت کی غیر موجودگی میں، میں اپنے دل کی حرکت سے آگاہ / باخبر تھا (مثلاً دل کی دھڑکن بڑھنے کا احساس، دل کی دھڑکن میں بے قاعدگی)	25
3	2	1	0	میں نے بے دلی اور مایوسی محسوس کی	26
3	2	1	0	مجھے احساس ہوا کہ میں بہت چڑچڑا ہوں	27
3	2	1	0	مجھے احساس ہوا کہ میری پریشانی حد سے بڑھ گئی ہے	28
3	2	1	0	جب بھی کسی بات نے مجھے پریشان کیا، اس کے بعد مجھے پرسکون ہونے میں دشواری کا سامنا کرنا پڑا	29
3	2	1	0	مجھے اس بات کا ڈر محسوس ہوا کہ میں کسی معمولی مگر غیر مانوس کام کی وجہ سے نکال دیا جاؤں گا	30

3	2	1	0	میں کسی بھی چیز کے بارے میں پرجوش ہونے کے قابل نہیں تھا	31
3	2	1	0	میں نے اپنے کام کے دوران مداخلت کو برداشت کرنے میں مشکل محسوس کی۔	32
3	2	1	0	میں اعصابی تناؤ کی حالت میں تھا	33
3	2	1	0	میں نے محسوس کیا میں کافی غیر اہم تھا	34
3	2	1	0	میں نے ایسی کسی بھی بات کو برداشت نہیں کیا جو میرے کام کو جاری رکھنے میں مداخلت کرتا تھا	35
3	2	1	0	میں نے خود کو خوفزدہ محسوس کیا	36
3	2	1	0	مجھے مستقبل میں کوئی چیز ایسی نظر نہیں آتی جس کے متعلق میں پرامید ہوں	37
3	2	1	0	مجھے محسوس ہوا کہ زندگی بے معنی ہے	38
3	2	1	0	میں نے خود کو بے چین ہوتے محسوس کیا	39
3	2	1	0	میں ان صورتحال کے بارے میں پریشان تھا جن سے میں خوفزدہ ہو جاتا اور خود کو بے وقوف بناتا	40
3	2	1	0	میں نے کپکپاہٹ محسوس کی (مثلاً ہاتھوں میں)	41
3	2	1	0	میں نے کسی بھی کام کے پہل کرنے میں مشکل محسوس کی	42

Scores for Depression, Anxiety and Stress are calculated by summing the scores of the relevant items:

Depression score

Q3 + Q5 + Q10
Q13 + Q16 + Q17 + Q21
Q24 + Q26 + Q31 + Q34
Q37 + Q38 + Q42

Total score =

Anxiety score

Q2 + Q4 + Q7
Q9 + Q15 + Q19 + Q20
Q23 + Q25 + Q28 + Q30 + Q36
Q40 + Q41

Total score =

Stress score

Q1 + Q6 + Q8
Q11 + Q12 + Q14 + Q18
Q22 + Q27 + Q29 + Q32 + Q33
Q35 + Q39

Total score =

Score Interpretation:

	Depression (D)	Anxiety (A)	Stress (S)
**Normal**	0 – 9	0 – 7	0 – 14
**Mild**	10 – 13	8 – 9	15 – 18
**Moderate**	14 – 20	10 – 14	19 – 25
**Severe**	21 – 27	15 – 19	26 – 33
**Extremely Severe**	28+	20+	34 +
